# Presenteeism Among Nurses in Switzerland and Portugal and Its Impact on Patient Safety and Quality of Care: Protocol for a Qualitative Study

**DOI:** 10.2196/27963

**Published:** 2021-05-13

**Authors:** Filipa Pereira, Ana Isabel Querido, Marion Bieri, Henk Verloo, Carlos António Laranjeira

**Affiliations:** 1 School of Health Sciences HES-SO Valais/Wallis Sion Switzerland; 2 Institute of Biomedical Sciences Abel Salazar University of Porto Porto Portugal; 3 School of Health Sciences Polytechnic of Leiria Leiria Portugal; 4 ciTechCare - Center for Innovative Care and Health Technology Leiria Portugal; 5 CINTESIS – Center for Health Technology and Services Research Leiria Portugal; 6 Department of Nursing Sciences Valais Hospital Sion Switzerland; 7 Service of Old Age Psychiatry Lausanne University Hospital Lausanne Switzerland; 8 Research in Education and Community Intervention Viseu Portugal

**Keywords:** healthcare, nurses, predictors, presenteeism, quality of care, frontline, managers, Portugal, Switzerland, patient safety, patients, safety, stress, emotion, knowledge transfer, acute care, long-term care

## Abstract

**Background:**

Nurses dispense direct care in a wide variety of settings and are considered the backbone of the health care system. They often work long hours, face emotional stress, and are at a high risk of psychosocial and somatic illnesses. Nurses sometimes fall sick but work regardless, leading to presenteeism and subsequent risks to quality of care and patient safety due to the increased likelihood of patients falling, medication errors, and staff-to-patient disease transmission.

**Objective:**

This study aims to understand presenteeism among frontline nurses and nurse managers in acute, primary, and long-term health care settings and to contribute to the development of future interventional studies and recommendations.

**Methods:**

A qualitative study based on online focus group discussions will explore the perceptions of, attitudes to, and experiences with presenteeism among frontline nurses and nurse managers. Using a pilot-tested interview guide, 8 focus group discussions will involve nurses working in acute care hospitals, primary care settings, and long-term residential care facilities in Switzerland’s French-speaking region and Portugal’s Center region. The data collected will be examined using a content analysis approach via NVivo 12 QSR International software.

**Results:**

The University of Applied Sciences and Arts Western Switzerland’s School of Health Sciences and the Polytechnic of Leiria’s School of Health Sciences in Portugal have both approved funding for the study. The research protocol has been approved by ethics committees in both countries. Study recruitment commenced in February 2021. The results of the data analysis are expected by September 2021.

**Conclusions:**

This present study aims to gain more insight into the dilemmas facing nurses as a result of all causes of presenteeism among frontline nurses and nurse managers in different health care settings. The researchers will prepare manuscripts on the study’s findings, publish them in relevant peer-reviewed journals, exhibit them in poster presentations, and give oral presentations at appropriate academic and nonscientific conferences. Regarding further knowledge transfer, researchers will engage with stakeholders to craft messages focused on the needs of nurses and nurse managers and on disseminating our research findings to deal with the issue of nursing presenteeism.

**International Registered Report Identifier (IRRID):**

PRR1-10.2196/27963

## Introduction

### Rationale

According to the United Nation’s 2030 Agenda for Sustainable Development, all countries should have a healthy, well-educated health care workforce with the knowledge and skills needed for productive, fulfilling work and full participation in society [1]. Inside health care systems, nurses are the primary providers of direct care, delivering vital services, and often considered the system’s backbone [2,3]. Undeniably, the SARS-CoV-2 coronavirus has emphasized that many health care settings are also workplaces where nurses face particular risks from occupational exposure to diseases and stress [4]. A perfect illustration of organizational presenteeism was seen during the COVID-19 pandemic in Australia: Hospital staff infected with SARS-CoV-2 while at work continued working for up to 7 days, even with respiratory symptoms [4]. The International Council of Nursing has confirmed that more frontline nurses were affected by SARS-CoV-2 than all the other health care professions combined [5].

Nurses often face difficult work conditions, including working long hours, overtime, and emotional stress: They are at a high risk of developing psychosocial and somatic illnesses [6]. Despite these poor working conditions, much of the absenteeism previously noted among nurses has been replaced by presenteeism [7,8]. Presenteeism may be described as the act of a health care professional who continues to work while sick or suffering from another condition that results in their underperformance at work [3,9]. One frequently used definition of presenteeism in nursing is the “act of being physically present at work when one should not be there” [10]. However, numerous studies of presenteeism have included identified etiologies as conditional for this behavior, such as “a physical presence at work when one should not to be there due to one’s health and well-being, a stressful work environment, a lack of work-life balance, or a sense of professional identity or obligation” [10-13].

Although it is widely recommended that nurses be in good health when providing health care to patients [1], in some cases and for numerous reasons, some sick nurses do not follow recommendations to stay at home and continue to work, leading to presenteeism [14]. The existence of presenteeism differs by sector, but it is more likely to occur among staff working in jobs with extensive interpersonal interactions with clients or patients [15]. There are many reasons why sick health care professionals might continue working. Nurses face pressures that contribute to presenteeism, including difficulties finding replacements due to workforce shortages, strong organizational-culture barriers, and professional-culture norms against taking sick leave [16]. Worries about presenteeism are not limited to potential loss of earnings; the lack of replacement staff; concerns about the resulting burdens on patients, clients, customers, co-workers; and a potential competitive disadvantage [14]. Presenteeism occurs not only when physically or mentally unwell nurses go to work but also when their level of awareness or responsiveness is compromised or when their emotional, behavioral, or cognitive engagement is diminished [11,12,17-20]. Nurses are 4 times more likely to exhibit presenteeism than other professions; yet, this threatens patient safety through increased falls, medication errors, and staff-to-patient disease transmission [21].

### The Concept of Presenteeism in Nursing

**Figure 1:**
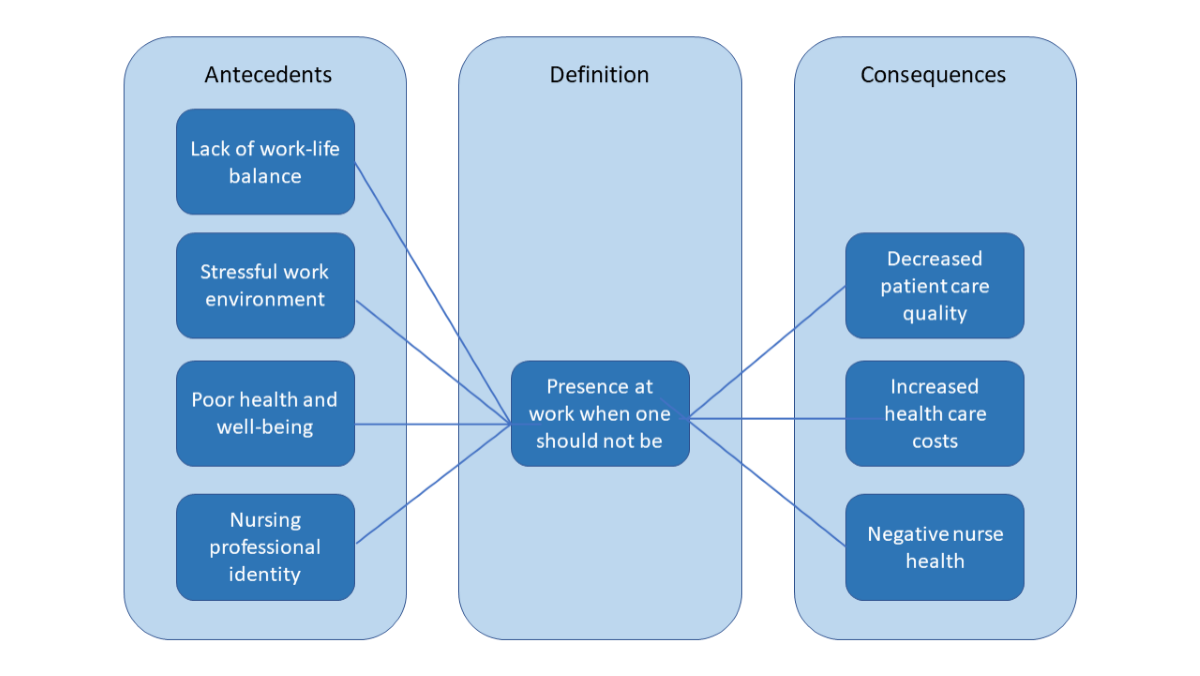
Conceptual model of presenteeism in nursing developed by Rainbow and Steeg [10].

The concept of presenteeism has been described across different professions, organizations, and work environments [9,17,22]. One multisector study of presenteeism found that nurses reported 3-4 times higher rates of presenteeism than employees in management positions, and they had the highest rate among the 42 occupational groups surveyed [17]. The concept of presenteeism in nursing has been related to the Quadruple Aim Framework (population health, patient experience, provider experience, and costs) linking optimizing nurses’ performance and their own health to harmful patient outcomes and increased health care costs [23,24]. Unlike factors such as nurse staffing ratios and nursing shortages, which impact care and costs, nurses’ behaviors are seen as potentially correctable in the short term and within organizations [19]. Figure 1 presents the conceptual model of presenteeism in nursing and the underlying relationships between presenteeism’s antecedents, definition, and consequences, by Rainbow and Steeg [10].

### Prevalence of Presenteeism

In 2016, Barbosa [25] identified a prevalence of presenteeism among Portuguese nurses of 91.4% in the previous month, more related to psychological reasons than to physical ones. Meanwhile, 32.9% of professional care workers in 162 Swiss nursing homes reported their own presenteeism during at least one shift in the month before being surveyed [26]. A very broad range of other estimates was also reported in a systematic review, where self-reported presenteeism in relation to an infectious illness ranged from 37% to 97% in the health care sector [14]. Although these rates varied considerably, even the lower end of this range is troubling, as it would probably have resulted in increased rates of transmission of infection in the workplace [14]. The heterogeneity of prevalence rates should be considered in light of different variables: (1) the sample characteristics (population and response rate), (2) the type of presenteeism studied (sickness, nonsickness, overall), (3) the variation in presenteeism recall periods (ranging from 7 days to 1 year), (4) the frequency and experience of episodes of presenteeism, (5) the threshold for presenteeism’s seriousness, and (6) the heterogeneity of measurement instruments [14].

### Consequences of Presenteeism

#### Decreased Quality of Care and Patient Safety

Several studies have demonstrated that presenteeism affects nursing quality and patient care outcomes [23,24,27,28]. High-quality nursing is expensive, but poor-quality nursing can lead to higher health care costs via potential adverse events, whether detected or not; increased hospital or intensive care unit lengths of stay; and even earlier death [23,28,29]. Unlike factors such as nurse staffing ratios and nursing shortages, which affect care and costs, nurses’ behaviors can be adjusted in the short term and within organizations [30-32].

#### Economic Costs

A systematic review by Kigozi et al [33] revealed that, on average, the cost of presenteeism comprised 52% of the total costs of the disease conditions investigated. The proportion of presenteeism was highest among employees suffering from rheumatoid arthritis, back pain, and insomnia. In 5 of the studies included in that review, the costs of presenteeism were greater than those for absenteeism, which was explained by the chronicity of the conditions investigated [33]. Although presenteeism has been associated with significant costs, losses from reduced productivity at work are rarely included in cost-effectiveness or cost-utility analyses. Ignoring these costs could significantly underestimate the true value of interventions that reduce nurses’ limited functioning at work due to illness [34].

#### Reasons for Presenteeism

Most studies have reported similar types of reasons that can be grouped into 3 overarching themes: organizational factors and working conditions, job characteristics, and personal reasons [14].

The *organizational factors and working conditions* explaining presenteeism resulted from internal organizational policies and the suggestion that working while ill was due to employees not being protected by paid sick leave or having no more available sick leave entitlement [31,35]. A culture of presenteeism in certain organizations shed light on the fact that this could become a social norm, embedded in the organization’s culture. Taking sick leave might even lead to disciplinary action [14,35,36]. Worries about losing a job were a major concern in many studies, whereas fears of getting into trouble, receiving a poor evaluation, being somehow penalized, and being anxious about job security were also reported [31,35,37]. Economic difficulties and the risk of unemployment may push nurses to presenteeism when they are ill or accepting excessive overtime [34,38,39]. Depending on the health care system, regulations concerning salaries, employment rules, and working conditions can reduce absenteeism and instead increase the incidence of presenteeism [34]. Presenteeism eventually leads to more health problems and a loss of productivity due to excessive working hours and feelings of insecurity; it can also develop into normal employee behavior, installing a culture of not missing work and working to meet the hierarchy’s service demands. The situation is worse among nurses who have chronic diseases and are more likely to practice presenteeism due to social pressures [40]. However, episodic conditions, such as allergic disorders, the common cold, and pregnancy, contribute to high levels of presenteeism among health care staff [41-43].

Some *job-related factors* also cause presenteeism. Nurses often have a strong work ethic and feel an obligation towards their patients and colleagues: Taking sick leave might jeopardize their reputations. A nurse’s professional identity is built on maturity and self-esteem reflected in their self-image. The professional function of a nurse is characterized by an elevated level of psychological commitment and sometimes expressed as the “super nurse phenomenon” [44]. This can result in many nurses working in health care settings being apprehensive about being covered for by agency or bank nurses, when available, and therefore reluctant to go absent or on sick leave [45,46]. High workloads may influence presenteeism because tasks might be left undone during an absence, creating fears of falling behind with tasks and having to make up for lost time on returning to work [12,47].

Some studies have demonstrated *personal reasons* for presenteeism [14]. A major one is that nurses did not want to burden colleagues with the extra workload resulting from their absence, and they often felt guilty about asking colleagues to cover their duties. Some nurses feared that colleagues would perceive them as weak and irresponsible if they were absent from work [48]. Another personal reason concerned the *financial stress* felt by nurses: They could not afford the loss of earnings inherent in taking sick leave as they needed to support their family [49].

A common theme running through the reasons given for presenteeism was nurses feeling that their illness did not meet the *threshold* of seriousness for taking sick leave and that it did not influence their capacity to carry out their duties. If they believed their illnesses to be noninfectious, then they supposed that they were not a risk to colleagues or patients, and they therefore chose to attend work [50,51].

### Relationships Between Sociodemographic and Professional Characteristics, Health Status, and Presenteeism

#### Sociodemographic and Professional Characteristics

Studies exploring *age* and *sex* as determinants of presenteeism have found no significant associations but should be considered inconclusive [14,52]. Nurses working in hospital settings had higher levels of presenteeism than those in long-term residential care settings, although without reaching the level for significance. No significant associations were found between presenteeism, *professional experience*, the number of *working hours* per week, or the type of patients cared for. Likewise, *job satisfaction* and the amount of work left undone if absent showed no significant associations with presenteeism. Nurses’ perceptions of the infection control measures in their health care settings were associated with presenteeism, with those who thought there was poorer control showing higher levels of presenteeism [14,53].

#### Nurses’ Health Statuses

Nurses’ health statuses and chronic conditions, such as asthma or diabetes, were not significantly associated with presenteeism. However, nurses whose otherwise healthy immune systems were weakened by illnesses such as cancer or immunosuppressant medication were more likely to report presenteeism. Numerous studies have found indications that presenteeism is associated with nurses’ past and intended work behavior with regards to having influenza [54,55].

### Measurement of Presenteeism

Presenteeism among nurses has been explored in numerous health care settings using quantitative, qualitative, and mixed methods research designs. The systematic review by Ospina et al [56] reported more than 20 self-administrated presenteeism instruments usable for different professions and work settings. Based on the COSMIN methodology [57], the instruments providing the strongest level of evidence are the 6-item Stanford Presenteeism Scale, the Endicott Work Productivity Scale, and the Health and Work Questionnaire [56]. To the best of our knowledge, there is a scarcity of qualitative research into presenteeism [44]. Most studies of nurse presenteeism have focused on the consequences for patients, linking it to increased rates of patient falls, medication errors, missed care, and changes in patient safety error reporting [58,59]. It is also possible that the consequences of presenteeism in nursing are different from those found in other industries or the patient consequences associated with presenteeism among other health care professionals. Nurses have not previously been asked to describe presenteeism's consequences but have instead completed retrospective self‐reporting surveys. Asking nurses what they perceive to be the consequences of their presenteeism will expand this body of research.

## Methods

### Aims and Research Questions

The present study aims to understand presenteeism among frontline nurses and nurse managers in acute, primary, and long-term health care settings and to contribute to the development of future interventional studies and recommendations.

To address known concerns about presenteeism and its impact on the quality and safety of care, the planned investigation should attempt to provide well-developed answers to the following research questions: What are frontline nurses’ and nurse managers’ perceptions of, attitudes toward, and experiences with the personal, professional, and contextual or organizational factors that lead to presenteeism? What are frontline nurses’ and nurse managers’ perceptions of, attitudes toward, and experiences with adverse health outcomes among patients resulting from nurse presenteeism? Are there different perceptions and attitudes about the reasons for presenteeism among frontline nurses and nurse managers?

### Study Design and Conceptual Framework

To gain more insight into nurses’ dilemmas resulting from all causes of presenteeism, our investigation will use a qualitative design and focus on participants’ perceptions, attitudes, and experiences.

**Figure 2:**
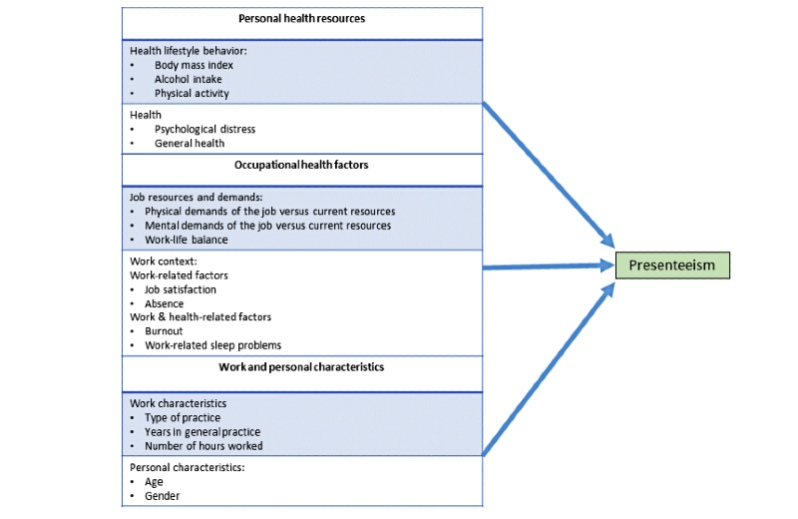
The study’s conceptual framework [60].

The study’s conceptual framework is based on prior work published by Pit and Hansen [60]. As an adaptive behavior aimed at meeting the demands of work or performance criteria during periods of impaired capacity due to ill health, 3 precipitating factors give rise to presenteeism, namely (1) personal health resources, (2) occupational health factors, and (3) work and personal characteristics (Figure 2) [60].

### Population and Settings

The research population will be composed of frontline nurses and nurse managers working in acute care hospitals, primary care settings, and long-term residential care facilities in Portugal’s Center region and Switzerland’s French-speaking region.

### Recruitment, Focus Group Discussion Procedures, and Data Collection

#### Participants

Eligible health care settings will be contacted by telephone or through a visit to determine their interest in participating in the study. A list of eligible nurses (frontline nurses and nurse managers) will be requested from the institution. Using a purposive sampling method, eligible participants will be recruited from the lists provided. A research team member will invite eligible participants to join the study. If the nurse agrees, an appointment will be arranged for them to respond to questions about a clinical vignette before participating in an online focus group discussion (FGD). The inclusion and exclusion criteria are listed in Textbox 1.

**Inclusion and exclusion criteria.**

**Inclusion criteria**

- Full-time frontline registered nurses with a bachelor’s or master’s degree or a PhD
- Full-time nurse managers
- Access to a computer, smartphone, or tablet with an internet connection

**Exclusion criteria**

- Less than 1 month’s employment in their current health care setting
- Student nurses

#### Procedure for Focus Group Discussions

FGDs will take place via videoconferencing software, but only audio will be recorded. A key advantage of this type of software is its ability to record and store sessions securely without recourse to third-party software. This feature is particularly important in research where highly sensitive data require protection. Institutional servers in each country will be used to ensure data protection and secure storage. To ensure that online discussions are manageable, FGDs will only include 6-10 participants. Before commencing data collection, participants will be asked to sign a written informed consent form, and confidentiality will be guaranteed. Participants will fill out a questionnaire about their sociodemographic characteristics. A URL link will be sent to participants by email so they will not have to download the program onto their own computer or mobile phone.

A total of 8 FGDs will be planned and run in Portugal and Switzerland, in public and private health care settings, depending on data saturation. The FGDs are expected to last 60-90 minutes each. The Portuguese research team will conduct 4 FGDs in acute care hospital settings: 2 among frontline nurses active on acute care wards and 2 among nurse managers. The Swiss research team will conduct 4 FGDs: 2 in long-term residential care facilities (frontline nurses and nurse managers) and 2 in community health care settings (frontline nurses and nurse managers).

#### Data Collection

Data will be collected via FGDs and clinical vignettes.

FGDs will collect data to investigate similarities and differences in the perceptions, attitudes, and experiences of frontline nurses and nurse managers. FGDs can be defined as semistructured discussions with stakeholder groups of 6-10 people that aim to explore a specific set of questions. Moderators often start an FGD by asking questions of general interest before asking more specific ones. Although participants respond individually to the researcher's questions, they are encouraged to talk and interact with each other. This technique is based on the notion that group interaction encourages respondents to explore and clarify individual and shared points of view [47-49].

Each FGD will start by presenting a situationally and culturally adapted vignette about presenteeism. This will be followed by a semistructured interview about presenteeism and the quality and safety of care. The interview guide will include the following topics based on the study’s framework: the management of presenteeism, the causes of presenteeism, its impact on the quality and safety of patient care, how colleagues deal with and perceive sickness presenteeism (practically, ethically, and socially), professional recognition by superiors and colleagues, and satisfaction with their professional quality of life. The semistructured interview guide for the FGDs will be pretested and refined in a pilot FGD involving 3-4 volunteers who meet our inclusion criteria (Multimedia Appendix 1).

The semistructured questionnaire and the clinical vignette have been built using the 3 concepts of personal health resources, occupational health factors, and job resources and demands [57].

To better understand the reasons for presenteeism among frontline nurses and nurse managers, each participant will receive an identical clinical vignette with open questions to see whether they understood the situation presented to them. A pretest phase is planned to test participants’ understanding of the clinical vignette’s content and the extent of the responses obtained from 3-4 volunteers meeting our inclusion criteria. The data collected during this phase will enable eventual modifications to be made to the vignette’s content or the wording of the questions to make them more straightforward.

### Data Analysis

Data will be analyzed according to good clinical research practices based on the dimensions included in the theoretical framework. Responses to the clinical vignette will be analyzed according to usual practices and respondents’ responses to the open-ended questions concerning the situations described. Descriptive statistical analyses will be performed using SPSS, version 27 (IBM Corp, Armonk, NY). These will describe the sample and establish the typical profiles of participating nurses.

Analysis of the data collected during the FGDs will be carried out via a qualitative content analysis approach [51-53] using NVivo 12 QSR International software. This analysis will enable a thorough description and examination of the causes and predictors of presenteeism, as well as their impacts on the quality and safety of care. A transversal reading of the interviews can be done at the end of the category analysis. This reading will allow transversal themes to be updated (occurrence of significant themes identifiable in the interviews) even if they are not linked to the pre-established categories. The results will be presented by category, and occurrences will be illustrated with significant examples, including verbatim quotes from the interviews. Following the analysis of each FGD, a second-stage analysis will compare findings across the groups. This will involve talks within the research team to refine the discussed themes and to develop higher-level themes, that is, grouping the open codes into meaningful conceptual categories.

To ensure study reliability, the researchers will be deeply involved in data handling (eg, transcribing, reading, and rereading the transcripts; conducting the inductive analysis) and will maintain transparency while they analyze the data subjectively. The research team includes 4 nurses involved in academic teaching and with expertise in clinical nursing practices (CL, HV, AQ, FP), as well as a research psychologist (MB). Data analysis will be guided by the COREQ guidelines [61].

## Results

The University of Applied Sciences and Arts Western Switzerland’s School of Health Sciences (HES-SO Valais/Wallis) and the Polytechnic of Leiria’s School of Health Sciences in Portugal have both approved funding for the study. The research protocol has been submitted for approval by both institutions’ ethics committees (Human Research Ethics Committee of the Canton of Vaud n° 2021-00071 and the Comissão de Ética do Politécnico de Leiria n° CE/IPLEIRIA/44/2020). At the time of writing, no participants had yet been recruited. Study recruitment commenced in February 2021, and the FGDs will be conducted from March 2021 to May 2021. The results of the data analysis are expected to be available by September 2021.

## Discussion

### The Study’s Expected Impact

Nurses’ services have been in increasing demand since the outbreak of the COVID-19 pandemic. It is vital to ensure that they have the resources to mitigate the impacts of this and new pandemics—when health care professionals face greater risks than usual. Forging closer working relationships between nurse managers and frontline nurses will also enhance their mutual understanding of risk factors and help to generate more effective ways of managing absences due to sickness and rehabilitation during a pandemic. This will require health care organizations to take a proactive approach to employee well-being and to make sure their policies encourage rest and recovery rather than promoting an “always on” or “work at all costs” culture.

We expect that the findings from our Swiss-Portuguese research partnership will contribute to a better understanding of where the thresholds for presenteeism lie. Our recommendations should lead to other multicenter studies aiming to develop effective measurement scales for presenteeism, as an essential first step to designing interventions that improve the health and well-being of nurses in their workplaces.

The proposed study is relevant because, to the best of our knowledge, there have been few investigations of health care institutions’ presenteeism prevention strategies. Indeed, these may not be well-developed at all because several studies have revealed that health care professionals are often the main victims of their own presenteeism (ie, it can compound their health problems), which can lead to a greater potential for making mistakes. Furthermore, the economic and financial demands of recent decades have led to the adoption of policies to contain health sector expenditure. This study will explore the causes of presenteeism—the fact that it entails costs far higher than absenteeism makes this investigation necessary—and aims to develop effective interventions to prevent or limit it [62]. We will collect frontline nurses’ and nurse managers’ perceptions of presenteeism and its impact on their work and the quality and safety of patient care from the point of view of their job function. This will provide us with information to formulate recommendations on reducing presenteeism to strengthen nursing and health care teams and to optimize the quality and safety of patient care. Researchers in both countries will supplement their analyses with contextual information (eg, policy, regulations, workplace management, employment conditions, geographic location) that reflects the results.

### Ethical Considerations

The research protocol has been submitted for approval to the research ethics committees of both the institutions involved. A research information form and a consent form will be given to each participant, specifying the study’s objectives, what participation will entail, and the measures taken to protect the participants’ rights and data. All participants will be kept informed about the study and will be free to withdraw their signed consent. They will also be asked for their consent to participate in FGDs and be audio-recorded (for transcription purposes) and video-recorded (although these will not be kept). Volunteers will receive no compensation for their participation. All the data collected will be treated confidentially, coded, and kept under lock and key for 20 years. Results will be presented in a way that respects participant confidentiality, and none will be identifiable either in presentations or publications.

### Methods of Disseminating Findings

Knowledge transfer will be appropriately considered and outlined in a dissemination plan focused on the needs of the audience that will use that knowledge. Researchers will also collaborate with knowledge users to craft messages and help disseminate research findings. Additionally, the researchers will prepare manuscripts and publish the study’s results in relevant peer-reviewed journals and present their findings via poster and oral presentations at appropriate academic and nonscientific conferences.

## Appendix

Multimedia Appendix 1 Interview guides.

## Abbreviations

FGD: focus group discussion
