# Supplementary material for: Presenteeism Among Nurses in Switzerland and Portugal and Its Impact on Patient Safety and Quality of Care: Protocol for a Qualitative Study
Source: JMIR Res Protoc. 2021 May 13;10(5):e27963. doi: 10.2196/27963 (PMC8160804; doi:10.2196/27963)
Supplement: Multimedia Appendix 1 [file resprot_v10i5e27963_app1.docx]

**Interview guides**

**Frontline nurses**

**Vignette:**

Take the case of Mary. By all accounts Mary was very bright and outgoing in high school. After graduating she started her activity as a nurse in a medical ward. Over the years she has reported difficulties in dealing with work overload, given the shortage of staff. She asked to change service because she had been in the same workplace for 8 years. Slowly, over time Mary become depressed. The depression was not diagnosed and treated and manifested itself primarily in insomnia, irritability, and overeating. Mary become quite lethargic and her supervisor was concerned over the decrease in productivity and in particular her lack of attention to detail. Through her inattention Mary injured herself at work which calls into question the safety of her care. She is now considered a cost to the system even though she has actually been a cost for many years. Helping Mary at this juncture may prove to be very difficult, since treating entrenched depression can be time-consuming. Also, returning her to the same work environment would likely exacerbate the problem and perhaps create a permanent disability. The workplace would need to change her duties or the way she does her duties in order to avoid repeating the situation. All too often we have looked to the worker to change rather than expecting workplaces to change.

**QUESTIONS**

1. Based on vignette what is presenteeism and how is it impacting your work?

1. Please describe any experience of coming to work while you were sick (circumstances, situations factors that best reflect your experience of attending work that day). If you have been at work when you're not at your best, how did you realize it?

Probing Questions: • What was happening at the time of your experience? • What led you to decide to go to or stay at work? • What was it like to be at work that day? • How did you feel on that day (tell me more about that feeling)? • Did others at work comment? • Did your employer or anyone else try to send you home?

1. If there have been times when you have been at work when you felt like you should not be, what are the main reasons why you were there? And the main consequences to yourself or others?
2. How would you describe your immediate work environment (organisational and psychosocial domains)?

Probing Questions: •Is your job recognized by superiors and colleagues? •Do you feel pleased with your job? •Do you have colleagues who suffer from presenteeism? • Has the pandemic covid-19 we are experiencing contributed to aggravate this phenomenon?

1. What nursing supervisors and managers of health institutions can do to control and reduce presenteeism in their organizations? What can front-line nurses do to prevent presenteeism?

**Interview guide**

**Nurse managers**

**Vignette :**

Take the case of Mary. By all accounts Mary was very bright and outgoing in high school. After graduating she started her activity as a nurse in a medical ward. Over the years she has reported difficulties in dealing with work overload, given the shortage of staff. She asked to change service because she had been in the same workplace for 8 years. Slowly, over time Mary become depressed. The depression was not diagnosed and treated and manifested itself primarily in insomnia, irritability, and overeating. Mary become quite lethargic and her supervisor was concerned over the decrease in productivity and in particular her lack of attention to detail. Through her inattention Mary injured herself at work which calls into question the safety of her care. She is now considered a cost to the system even though she has actually been a cost for many years. Helping Mary at this juncture may prove to be very difficult, since treating entrenched depression can be time-consuming. Also, returning her to the same work environment would likely exacerbate the problem and perhaps create a permanent disability. The workplace would need to change her duties or the way she does her duties in order to avoid repeating the situation. All too often we have looked to the worker to change rather than expecting workplaces to change.

**QUESTIONS**

1. Based on vignette what is presenteeism and how is it impacting your activity as nurse manager/supervisor?
2. How do you view presenteeism among the nursing staff?
3. Why nurses find helpful in remaining at work or returning to work when they have health problems? What are the main reasons and consequences?
4. What implication does have this behaviour for patient safety and quality of care?
5. What chances have nursing supervisors and managers of health institutions to control and reduce presenteeism in their organizations? What decisions or actions have you taken in this regard?
